# Science of Practice Building

**Published:** 1910-01-15

**Authors:** James Wood Pogue


### SCIENCE OF PRACTICE BUILDING.

JAMES WOOD POGUE.

I am going to talk about the Science of Practice Building especially to dentists. I know dentists as a body, and all professional men, doctors, lawyers and so on, have to win success, but professional men have a certain pride which prevents them from getting after business; but there is a right and scientific way to build practice.

It is necessarily based upon identically the same principles as the science of business building in the ordinary business world.

I am well aware that the members of the various professions hold themselves somewhat apart from the average business man. That idea has been current to a degree in the past but is becoming less current to-day, because all men are recognizing more and more that business is a profession, and members of the recognized professions are likewise understanding that every man must in some way go out after the buying public.

Professional men are compelled to-day to study three things: First, how to develop the power and skill that the public want; second, how to bring to yourselves that portion of the buying public in need of your services; and third, to negotiate a mutually agreeable basis upon which you can render services and they receive them. That is identically the same principle which I am operating upon, which all high grade business men are operating upon to-day.

In other words, the central point of all your life is that of service. First, the power to render service; second, the opportunity to reach those who wish the service rendered, and, third, to reach a basis of compensation.

I wish we had here as many of the individual students of dentistry as we could get together in the State of New York, so these ideas might percolate into their minds to the end that they might reach the highest success in their profession. I have known of some men coming to want before they could get started; and I have known others who never did get a good start.

What every man looks for is that he may be able to attain in his profession something that we call success. And that success, as far as I know the dental profession, does not consist of some high ideal in some country far beyond the clouds where no interchange of cash ever takes place. By success as a dentist you mean, first, to be able to gather that kind of skill and power that will be of service to the public; next, to be able to persuade a sufficient volume of the public to come to your office and receive your services, to pay you for all the long years you have spent in perfecting yourself to render those services. And if you are able to persuade them to come, then you must be able to persuade them to return desirable money remuneration for your service.

We find in the investigation of business life and otherwise that all business success is based upon the volume of profitable trade one can secure, and the emphasis is upon

*profitable.* Some tradesmen as well as some dentists do not get profitable trade. What we want and must have is profitable trade—that which returns the cold, hard cash.

You have my subject stated as the Science of Practice Building. We do not want simply to build up a large practice. We can get all the practice we want, but we must have profit along with the practice.

Sometimes I meet people who tell me that they do not care anything at all about money. I have never known but three classes of people who did not care anything about money. First, those who were mentally unbalanced; second, those who were dyspeptics; and third, just plain liars. Those are the only three classes I know of that do not care about money.

There is only one way to get profitable trade, and that is by inspiring confidence in the buying public; not confidence only in your theory, but they, also, must have confidence in you. Confidence is always placed in a person, never in a thing, in the first instance.

Therefore, what you and I are to do if we build success is to inspire confidence, and we do that through our personality. Anything that enters into your personality or mine, which tends to destroy or prevent confidence, prevents our success in a like degree.

Every man in this room who is meeting the buying public knows that the one who is able to inspire the most confidence in people may not be the most skillful, but he will get the most profitable trade. Have you not seen that more than once? Has it not made you feel a little sore sometimes? We want to build that kind of personality which inspires confidence, and secures profitable trade, which insures success; that is thoroughly reasonable and scientific. In that display room out there they say to us about each thing, "This is thoroughly scientific." Every product in that room is supposed to represent (and I have no doubt it is true) the highest degree of scientific excellence, which is just the excellence demanded in practice building.

I am going to say something you may not like. During the last twenty-five years science has practically exhausted itself in preparations for the preservation of the teeth, in the proper understanding of how to treat the teeth, in the best mechanical appliances to use in drilling the teeth, and so on. And men have exhausted science in order to perfect themselves in the use of those appliances for the benefit of the public; but, gentlemen, I want to say to you in all frankness that the most of those who are using those appliances have not begun to expend upon their own persons and personalities anything like the scientific intelligence to perfect themselves, whereby they may be able to convey to the buying public that kind of confidence which brings the buying public to them. Nothing like the science has been expended upon yourselves that has been expended upon the mere appliances of your profession. There is the crucial point in your work. Stick a pin there (pointing to Personality on a chart, on which he had made a pyramid of the foundation stones of success. It read this way:

Success<br>

Profitable Trade<br>

Confidence<br>

Personality<br>

Character; Health<br>

True Education).

How are you going to build that kind of personality? By building character—sterling character; health, robust health! and one is just as important as the other! They are the essential factors of true education.

Gentlemen, you know a great many things about your profession, but here is something that few men in the professional life of this country have yet mastered. In the investigation of these sciences in the business world we have found that in every trade transaction there are four elements—and the first element is the salesman.

You may say, "I am no salesman." But are you not selling service and taking pay for it? If that is not so we will call it all off and I won't talk any more. The first element in the transaction, gentlemen, is the salesman.

(Here the lecturer made another column on the chart, which read:

Salesman
Customer
Service
Sale).

As professional men we are to study how to perfect the *salesman*, for here is the basic principle. The more man you build the more business you build, and the better business you build. You and I in these transactions have a right to build the manhood of our humanity to the highest possible degree, and in building that manhood we must build: Body! Brains! Soul! Will!—and, if you leave out either one you ruin the others.

The second element is the customer—something we do not study half enough. Let me ask any of you gentlemen as professional men in seeking to render service to the buying public, have you ever studied how to read the nature of that public? Is the most skillful man in this room in your profession the man who most absolutely desires to place at the disposal of the long-suffering public all the possibilities of his skill? If so, he should study how to read men in order to bring them to him, because—I say it reverently—God knows that there are a lot of people practicing on other people's teeth that the public could do very well without. And the men who know how, ought to see to it that they are the ones to whom the buying public comes. That is fair to the public as well as to ourselves. So we should study the customer, his needs and nature, and how to reach him most effectively.

The third element in the sale is the article or service to be rendered; in this case, for the article, we render service and we should understand that service so as to be able to advise the customer, when he is buying, just what is best for him in a given condition.

And the last element, gentlemen, is the sale (pardon the word). You tell me you are not peddlers, nor agents; but, notwithstanding, you and I are selling service, and he profits most who serves best, and unless you render service you shall not be benefited yourselves nor benefit others.

You may consider all the dentists that practice in New York for the next ten years—and that will be a few—and all the suffering public with aching teeth and swelling jaws, and you may be able to render all the best service in the world, unless the sale takes place all that which goes before does not amount to a snap of the finger. I care not what your ability or skill, or the possibilities of your life, to the buying public, unless the service is rendered there is nothing doing.

"Almost" sales never profit us anything, and they never profit the public. The people who come to your door and go away, never put any money in your pocket or bring advantage to themselves, profit you nothing.

We are to study also:

Character Building
Health Building
Character Reading
Business Logic
Business Profit.

To promote the four elements described above we need to undertake these studies: Character building, the building of ourselves. We can all build our characters, I am not talking just about being good—we mean much more than that. Goodness is only one element of character.

We need to study the science of life, of Health Building. I have been to a dentist's office and had so foul a breath blown in my face that it seemed that I could hardly sit in the chair.

The third science is character reading, how to read the other person's character and treat him according to his nature. You cannot handle everybody in the dentist's office just alike. How many of you ever sat down and studied the different ones and how to treat them? And yet it is so easy to read people if you just learn how.

Then comes the science of business logic. I once asked my dentist for something I really did not need and the dentist should have told me better, but he did me a life-long injury instead, for which I have suffered much.

You gentlemen as professional men need to study the science of administering your service so that the buying public will get what is best for it to have, for few of them know what that best really is.

The next essential study is business psychology—how to bring your business transaction to a close. The one thing the buying public wants of you is value in return for fee. The one thing you want of the public is fee in return for value. How often you have rendered value and did not get the fee!

The one thing the public wants is to have value in return for value. What is my measure of value as a dentist or an employee of the public? For that is what I am. Let no man be ashamed of being called a servant of the public. "He who would be the greatest among you, let him be the servant of you all," said One long ago, who is second to no man that earth ever held.

What is the measure of my value? It is not the number of years I spent at college, nor the number of hours that I spent over the tooth—nor the gold that I pounded into it —not at all. The real measure of my value is the amount of supervision that I really need on my work each day. What is the occasion or meaning of supervision? The occasion is error. The man who never made a mistake never made much of anything else, but we have no right to continue to make the same mistakes we have made before. How many kinds of error are there? Only two—mistakes of omission and mistakes of commission: doing the things we ought not to do and leaving undone the things we ought to do. You and I sometimes do that which we ought not to do, but more often the things we ought to do, we do not. The things we ought to do and do not are the things that make trouble. If you could eliminate all errors from your work, thereby doing away with the necessity of supervision, you would render the perfect value to the buying public.

What is the occasion of error? Something in us, gentlemen, which we may term a negative. There are fifty-two great negatives making for failure in the lives of men and ninety-eight per cent. of men are comparative failures. In the lives of men making a success there are fifty-two important positives, and only two per cent. of them build these to a marked degree. Nothing is small that makes for failure or that makes for success.

In Boston Harbor, in the summer of 1907, I had my own motor boat. I had never run a boat myself alone before, but after a little practice I grew somewhat venturesome, and took my boat and family and we went seven miles out to sea. Toward evening I said, "We will go home," and I turned the boat to start back home, for the harbor, and all of a sudden the old thing quit running. I could not see anything wrong. Gallons of gasolene, battery all right, everything looked right, but not an ounce of power! Finally my boy, who had had more experience than I, found a little screw, about the size of the lead in a pencil. He said: "I don't know as this is of any consequence, father, but you might tighten it up." So I did, with the point of my knife, and immediately I had all the power I wanted. How many, many men you and I know who are seven miles to sea and night coming on and not an ounce of power! Nothing much is the matter. Just a little screw loose—that's all—and if they had somebody to take the point of a knife and tighten it about one and one-half times they would be all right.

I will tell you about a professional man in Boston, a Harvard graduate, who killed his success in life. There was nothing wrong with him, one of the loveliest men I ever knew. He had an invalid wife for ten years, and he waited on her like a baby, and I never heard him grumble, but he was a failure. Because he had an offensive habit. He would come right up close to you and snuff, snuff, and he would keep it up until he would about set you crazy. If you have a habit like that, lose it.

You and I want to succeed, and we must get rid of the negatives. They may be ever so little, but remember that one little screw or tap loose in an automobile or boat will kill every ounce of power. Tighten up, men, tighten up! You have the most perfect piece of machinery that the most perfect Workman has ever been able to turn out. There is a reason why some of us are not successful. Are you brave enough to face the reason? Cassius to his friend Brutus said a long time ago, "The fault is not in our star, but in ourselves that we are underlings." If we want to find the cause of trouble, let us get a looking-glass and look straight into it, and we will find it there.

In the building of business success you and I are to take up a system of true education. There is much education that is false. My son wanted to go to school in Boston after he had been in the West, and he said: "They have a half dozen courses down there, and I don't know what to do." I asked him what they said, and he answered, "One fits you for this and another for that, and another fits you for life, and another fits you—" I stopped him right there and told him to take the course which would fit him for life.

In the system of true education the first thing to develop our bodies, and that to the very highest possible condition of physical vigor. We owe it to ourselves and to our Creator. A lot of people think religion consists in having a poor, sickly, pale body and a weak voice, but genuine religion consists in a strong, robust body, vigorous and active, and so does genuine business.

First, build a good strong and healthy body for your work. There are four things in body building: strength, activity, symmetry, endurance. You know how much strength it takes to extract a tooth, but do you know the great blessing to the patient in your chair of that strength that is magnificent if it is gentle?

Activity, oh, the people I know who are losing out in life because they are always just thinking about getting ready to go somewhere and do something. No real action. Just *thinking* about getting *ready* to go somewhere and do something! Do you ever think about the other fellow, waiting while you get ready? Dying at the stake does not compare with it. Get through with it! Do it now! And do it right! If you have not that motto up in your office, put it up.

Symmetry and endurance. The average man is using only one-fourth of his physical possibilities and one-tenth of his brain possibilities. The average brain shows only only one-tenth of the cells ever developed at all. There are a lot of dentists that are not getting anywhere near one-tenth of their gray matter to working. Get the balance working. The public needs it so badly, and there is such a chance for the fellow who gets even two-tenths working; he has so little competition, you see.

The brain consists of four powers; first to know. How do I know? All that I know is the product of sensation. I only know what I have seen, heard, tasted, smelled and felt. The more perfect my senses the more perfect my sensations and intelligence. As I build my senses and am able to see the condition and the needs of the patient, I thereby build my power for service and also build the values that I receive and give. And it is true, more true than you think, that many men handling nervous patients drive people just as far away from them as they can get, because they have no sensation, no idea of what the patient is undergoing.

To observe. Just think what some professional men do. I do not suppose you ever did anything like this. But a lady died in Philadelphia not long since who had been operated on seven years ago by two eminent surgeons in America. She had the incision sewed up and it continued sore, and she went abroad and all over Europe, and after much suffering she died the other day, and then, wishing to know just what the conditions were, the family consented to an examination. They cut the poor dead body open and found inside a pair of forceps that the very notable and distinguished surgeon had left there. And this occurs more times than is known. There are things done on that order, and you and I need to develop the powers of observation, which prevent such mistakes, and along with these the power of memory, the power of imagination and the executive powers.

And the third thing is to build the soul powers of our lives.

Referring back to the dentist, of whom I spoke, I remember him so well, not simply for what he did, but for what he was. When he had done his best for me—and his best was second to none—and I would rise from the chair and get ready to leave the office, he would come and give me his hand and say, "Good-by, sir, I am very sorry you had to suffer so." That was just a little touch of brotherly kindness. You can make a man feel that you are worse than a Wall Street shark when he is in your chair, but just a little touch of kindness does help so when the agony is over. Your patient will suffer, and I know how he suffers. Nobody suffers more than the dentist's patient. Remember, gentlemen, that being a professional man does not mean necessarily that a man shall swell up and strut, and think he is above feeling and such things. It is not right that any professional man should be lacking in feelings of kindness, especially the dentist. It is unfair to the profession in the first place, and unfair to the public in the second place. Preeminently a dentist should build his soul powers.

Then there is the power of faith. I want to render service—some see the suffering of the patient and do not have sufficient faith in their own judgment to go on and do what they know needs to be done. They cannot go on and do the necessary thing for lack of faith. We need faith in ourselves, faith in others, and faith in the One over and above all.

There is many a person whose life has been saved or shortened by the treatment of their teeth. You are the conservators of the best and highest interests of the public or you can be the purveyors of much that is bad.

I have been more thankful than I can express to the dentist in Boston, who, when my little girl had to go and have her teeth filled, was so kind to her—as kind as if she were his own little sister. I could not pay him for that, gentlemen. You might hurt me and I would not resent it, but if you hurt my little girl I would resent that; but if you are kind to her I will be kind to you and I will send others to you. By this soul power of kindness you attract me and all men. If you want to build your practice do it not only by building the brain and the grip of the hand, but by building the breadth of soul that can be strong and yet be kind.

Build also faith in God that gave the skill, that gave the power, that gave the privilege of service; for, gentlemen, I know nothing this side of heaven greater than the privilege of serving my fellow men. And along with that, genuine ambition to go on and climb to the top of our profession, willingness to pay the price for climbing—for climbing always costs. Oh! the men I know who got their skill and practice, and kept practicing and continued to practice—who never did anything more. They have no ambition to climb. They never inform themselves, and never study the things that make for the good and benefit of all, thereby most benefiting themselves.

We are to develop ambition as a soul power, and this is just as legitimate as any other power, and with that love and kindness toward everyone we touch, knowing that no service is good service unless two people are satisfied—the buyer and the seller, the one who serves and the one who receives.

The next soul power is reverence, which is preeminently needed in the lives of professional men. I do not care to go into the other side. Remember this: If you as professional men would render the highest service and build the highest possibilities in your own life, cultivate reverence. Reverence for what is clean, reverence for what is pure, reverence for what is holy. And see that your life is as the life of the purest that lies there in your chair to receive service at your hand. See to that, men, because it is important both for you and for those you serve.

These are the things you and I are to study, recognizing that life and the gift of life is in itself a privilege; and in addition to life, the privilege of service is the greatest thing man can possibly enjoy. And you and I in the profession which is ours should remember this: that the great thronging mass of humanity know little of the possibilities that our lives have for us. They have enjoyed little of the opportunities we have had. They see little of the future possibilities we see ahead of us. You and I as part of the educated class, walking through this world, will achieve the highest success and receive the highest financial reward—as every other kind of reward—by conserving the highest interests of humanity while we serve.

I thank you for the privilege of meeting you, and for the pleasure of witnessing to your service, and for those who stand ready to serve, as men whose skill is needed to render the highest possible value for the least possible pay that is right and proper that they should receive as men for God's service.—*Dental Digest.*

